# Why Muslim women in Northern Ghana do not use skilled maternal healthcare services at health facilities: a qualitative study

**DOI:** 10.1186/s12914-015-0048-9

**Published:** 2015-04-28

**Authors:** John Kuumuori Ganle

**Affiliations:** Department of Geography and Rural Development, Population, Health and Gender Research Group, Faculty of Social Sciences, Kwame Nkrumah University of Science & Technology, Kumasi, Ghana

**Keywords:** Northern Ghana, Maternal health, Maternal healthcare access, Religion, Islam, Muslim women

## Abstract

**Background:**

Muslim women are one sub-population in Ghana among whom the rate of skilled maternal health services accessibility and utilisation is very low. However, there are no studies in Ghana that explore the maternity needs and care experiences of Muslim women, and why they do not utilise maternal healthcare services at health facilities. The purpose of this paper is to explore the maternity healthcare needs and care experiences of Muslim women and the barriers to accessing and using maternal health services.

**Methods:**

Qualitative research was conducted with 94 Muslim women in three communities in northern Ghana between November 2011 and May 2012. Data were analysed using the Attride-Stirling’s thematic network analysis framework.

**Results:**

Findings suggest that although Muslim women do want to receive skilled care in a health facility, they often experience difficulties with accessing and using such services. These difficulties were often conditioned by a religious obligation to maintain bodily sanctity through modest dressing and the avoidance of unlawful bodily exposure or contact with certain people including male or alien caregivers. Other related access barriers include lack of privacy, healthcare providers’ insensitivity and lack of knowledge about Muslim women’s religious and cultural practices, and health information that lacked the cultural and religious specificity to meet Muslim women’s maternity care needs.

**Conclusion:**

Maternal healthcare services designed to meet the needs of mainstream non-Muslim Ghanaian women might lack the flexibility and responsiveness to meet the unique maternity care needs of Muslim women. Recommendations for change include cultural competence training for healthcare providers and cultural/religious matching to meet Muslim women’s care needs and to enhance their care experience.

## Background

Increasing the proportion of women who receive skilled maternal healthcare services, especially skilled attendance at delivery, is one of the most important policy actions needed to reduce maternal mortality in low-income countries [1,2]. While facility births have gone up dramatically in some settings in the last five years or so, some women still do not have access to health facilities and skilled birth attendants in many countries in the sub-Saharan African region where the burden of maternal mortality is relatively high [2-5]. For instance while in East Asia and the Pacific as well as in Latin America and the Caribbean, about 9 in 10 births occur in health facilities with a skilled birth attendant, in sub-Saharan Africa only about half of births (46%) are delivered in a health facility with a skilled birth attendant [6].

Like most countries in Africa, Ghana is one country in which for the majority of women, the experience of pregnancy and childbirth can be a fearful one [2-5]. In 2010, the WHO estimated that Ghana’s maternal mortality ratio (MMR) was 350/ 100, 000 live births [7]. Maternal mortality, which accounted for 14% of all female deaths, was the second largest cause of female mortality in Ghana [8]. Despite the fact that Ghana has since 2003 implemented a new maternal healthcare policy that provides free maternity care services in all public and mission healthcare facilities, recent survey data suggest that only 55% of women receive skilled assistance during delivery or postnatal care following delivery [9]. The survey also suggests that more than 45% of births still occur at home and elsewhere without any form of skilled care (i.e. without the assistance of an accredited health professional – such as a midwife, doctor or nurse – who has been educated and trained to proficiency in the skills needed to manage normal or uncomplicated pregnancies, childbirth, and the immediate postnatal period and in the identification, management, and referral of complications in women and newborns). This poor maternal health situation is compounded by widespread access inequalities between different socio-demographic groups across the country [2,10].

Muslim women are one sub-population in Ghana among whom utilisation rates of skilled maternal health services are particularly low [2,9]. Previous studies in Ghana found that women professing the Islamic religious faith were less likely to use antenatal care services, take all two doses of tetanus toxoid vaccine, choose a health facility as a place of delivery, and use postnatal care services as compared to Roman Catholic, Presbyterian, Methodist, and Pentecostal congregants [2,11,12]. Although a number of previous studies have hypothesised that this situation could be related to the religious beliefs and practices of Muslim women [2,5,12], there are no qualitative in-depth studies that examine the maternity care needs of Muslim women and what it is about Muslim women that makes them less likely to use skilled maternal health services. This, indeed, is reminiscent of Reimanova and Gustafson’s observation among immigrant Muslim women in a Canadian context that ‘to date, religious diversity in general, and Islamic religion in particular has been neglected in research on women’s maternity needs and access to care’ [13]. The purpose of this paper is to explore the maternal healthcare needs and care experiences of Muslim women and the barriers to accessing and using maternal health services in the Central Gonja District of the Northern region of Ghana**.** The choice of the Central Gonja District for this research was based on its poor maternal health outcomes and the high concentration of Muslim women in the area. While it is important to identify the maternal healthcare needs of all women to improve their health, studies that contribute to a better understanding of the maternity health needs of Muslim women and the barriers Muslim women face in accessing and using skilled maternal health services are particularly important for building more inclusive maternal health services that could improve equity in access, and propel progress towards the MDG-5 goal of reducing maternal mortality ratio by three-quarters between 1990 and 2015.

### Conceptual framework

Access to, and use of, maternal health services depend on a large variety of factors, including the nature of care offered such as cost and quality, and the characteristics of the population being served such as their wealth, education and cultural perceptions [3,14]. Therefore, no single framework might be sufficient in providing explanation to all the conditions that stimulate or hinder maternal health seeking among Muslim women in Northern Ghana. As this study was a formative one, the focus was on searching and discovering the relationship between religion and maternal health access among Muslim women. To articulate how the Islamic religion might influence Muslim women’s access to and use of modern maternal health services, this study adopted a theoretical framework that previous authors have called the ‘religio-cultural thesis’ [12,15,16]. The religio-cultural thesis is based on the notion that the philosophical ideals, norms, values and practices of various religious groups by themselves might influence maternal health accessibility and utilisation behaviour [12,16]. This conception derives, in part, from the notion that religion often claims a strong therapeutic component, and in some respects, dictates acceptable medical intervention [12]. The potential for religious beliefs and practices to exert some influence on behaviours and attitudes regarding use of modern maternal health services cannot be underestimated in the African context given the significant role religion plays in the social organisation of many African societies [16]. Under this religio-cultural framework thus, it is expected that Islamic religion will shape many aspects of Muslim women’s reproductive health seeking behaviours, and that independent of other factors, access to and use of maternal health services will be influenced by Islamic religious beliefs and practices.

The argument that the Islamic religion might be relevant to an understanding of Muslim women’s ability to access and use skilled maternal health services in health facilities in Ghana draws on previous research by Reitmanova and Gustafson, which identified the Islamic religion as shaping many aspects of Muslim women’s reproductive health seeking behaviours [13]. For instance, upon entry into the healthcare system, Muslim women were found to experience difficulties with getting *halal* food within health facility settings. Similarly, many healthcare providers were found to lack adequate understanding of the religious requirement for Muslim women to dress modestly by covering up their bodies. These difficulties discouraged a number of Muslim women from seeking skilled birthing services [13].

In applying the religio-cultural framework to examine why Muslim women do not use modern birthing services in Northern Ghana, the study followed the ‘appreciative inquiry approach’ [17,18]. Appreciative inquiry is about the co-evolutionary search for the best in people, their organisations, and the relevant world around them [17]. In its broadest focus, it involves the art and practice of asking questions that strengthen a system’s capacity to apprehend, anticipate, and heighten positive potential [18]. The use of the appreciative inquiry approach in this study enabled exploration and description of both positive and negative elements within Islamic communities, and the identification of internal capacities, strengths, and activities Muslim women deemed effective for promoting access to and use of maternal health services in health facilities [17]. With appreciative inquiry, the researcher was also able to explore and amplify what works and the successful elements and experiences that could inform learning and change without solely focusing on problems. This is particularly important because both previous research and promoters of maternal healthcare access among religious groups more generally, and Islamic religious groups in particular, have tended to focus disproportionately on problems and negative attributes that discourage use of skilled birthing services.

## Methods

### Research design

The qualitative data reported in this paper are part of a larger, original study that the author conducted to examine the effects of Ghana’s free maternal healthcare policy on women’s maternity care seeking experience, equity of access, and barriers to accessibility and utilisation of maternal and newborn healthcare services. The design of this larger study involved analysis of a nationally representative retrospective household survey data in combination with qualitative exploration using focus group discussions (FGDs), individual interviews (IIs), key informant interviews (KIIs), case studies and structured field observations. This paper focuses on, and reports findings from a part of the qualitative component of the study, which explored why Muslim women in northern Ghana do not use maternal healthcare services at health facilities.

### Study context

Empirical research was conducted in the Central Gonja District in the Northern Region of Ghana between November 2011 and June 2012 in 3 communities. Table 1 shows some of the characteristics of the study communities.

**Table 1:** **Basic characteristics of the study communities**

**Characteristic**	**Community**			**Entire Central Gonja District**
	**Tidrope**	**Mpaha**	**Buipe**	
Population (2010)	1,025	4,126	8,735	87,877
Number and type of health facility (2010)	None	Health centre (1)	Hospital (1)	Hospital (1), Health centres (5), CHPS zones (11)
Number of medical doctors (2010)	0	0	1	1
Number of midwives (2010)	0	0	2	9
Number of general nurses (2010)	0	1	3	6
Number of community health nurses (2010)	0	5	14	37
Number of pharmacists (2010)	0	0	0	0
Number of dispensary technicians (2010)*	0	1	1	2
Number of community based surveillance volunteers (2010)	1	2	11	518
Distance to nearest health facility (km)	13	2	1	n/a
Doctor to patient ratio (2010)	1: 110,576			
Nurse to patient ratio (2010)	1:2,572			
Number of maternal deaths (2010)	n/a	2	25	28
Number of under-5 deaths (2010)	n/a	10	14	25
Four plus ANC visits (%) (2010)	n/a	34	33	29.8
Skilled delivery (%) (2010)	n/a	18.1	22.2	13.4
PNC coverage (%) (2010)	n/a	56.4	44.3	33.6

*These are middle level healthcare personnel trained to dispense drugs at healthcare facilities where there are no qualified pharmacists.

According to Ghana’s 2010 Population and Housing Census, the district has an estimated total population of 87,877, of which 44,017 are females [19]. The population of the district is predominantly rural - 90% of the population is located in rural communities with populations of less than 500 people [19]. While the distinctions between those believing in African traditional religion and Islam/Christianity are not usually clear-cut, there are three main religious groups in the district, namely Islamic (70%), Christian (18%) and traditional African religion (12%) [20]. Agriculture, including animal rearing and fishing, is the main occupation of the people - it employs about 90% of the labour force [20].

At the time of this research, about 79% of the district female population (aged 6 years and above) had never been to school [20]. Women of reproductive age (i.e. 15–49 years) constituted more than half (22,316) of the district female population [20]. Under-5 mortality was estimated to be 181/1000 live births, compared to the regional and national figures of 155/1000 and 109/1000 live births respectively [21]. While data on maternal mortality was not readily available, the district had one of the poorest records in terms of access to skilled birthing services. For instance, in 2009, 2010, and 2011, skilled birth attendants attended only 14.4%, 13.4%, and 24.4% of births respectively [21]. Similarly, although the three study communities showed variable levels of performance on maternal health indicators, access to antenatal, delivery, and post-delivery services in all these communities were below the national averages (Table 1) [21].

### Ethics

The University of Oxford Social Sciences and Humanities Inter-divisional Research Ethics Committee (Ref No.: SSD/CUREC1/11‐051), and the Ghana Health Service Ethical Review Committee (Protocol ID NO: GHS-ERC 18/11/11) gave ethical approval for this study. In addition, informed written and verbal consents were obtained from all research participants. To minimize any potential harm to research participants, their identities have been anonymised in the analysis and reporting of the study results.

### Research participants

Participants comprised 94 Muslim women who were either pregnant at the time of this research or had given birth between January 2011 and May 2012. Table 2 shows the socio-demographic characteristics of participants.

**Table 2:** **Socio-demographic characteristics of participants**

**Characteristic**	**Number of women**	**Percentage**
**Age (yrs)**		
≥20	21	22.7
21-25	26	27.6
26-30	25	27
31-35	9	9.7
36-40	4	3.8
41-45	1	1.1
Don’t know	8	8.1
**Highest level of education**		
None	57	60.7
Primary	19	20.4
Middle/JSS	16	17.3
Secondary+	2	1.6
**Occupation**		
Farming	38	40
Petty trading	21	22.7
Housewife	21	22.7
Head dresser	6	6.5
Seamstress	7	7
Teacher	1	1.1
**Marital status**		
Married	64	69.1
Widow	5	4.9
Separated	9	9.2
Single	16	16.8
**Type of marriage**		
Monogamous	51	54.7
Polygamous	43	45.3
**Number of children**		
None	8	9.2
1-3	51	53.5
4-6	35	37.3
**Total number of pregnancies ever had**		
1-3	54	57.3
4-6	33	35.1
7-9	7	7.6
**Age at first pregnancy**		
15-20	36	38.4
21-30	50	53.5
Don’t know	8	8.1
**Place of last delivery**		
Home	63	73.3
Health facility	23	26.7

The ages of these women ranged from 15 to 45 years. Consistent with the district female population, majority of participants had no formal education. Most participants were also engaged in subsistent farming and petty trading in agricultural commodities. A few were however engaged in diverse occupations such as hairdressing, dressmaking, and shea butter and groundnut oil extraction. Several of the women were in polygynous relationships. The majority of the women also had between 3 and 5 children.

### Sampling and recruitment

The three study communities were purposively selected to capture a diversity of social and health situations that were largely representative of the district. The first community (Buipe) was selected to represent urban communities; the second community (Mpaha) was chosen to represent rural communities with health facilities; and the third community (Tidrope) was selected to represent rural communities without any health facilities. A mix of purposive and simple random sampling techniques was however used to select individual research participants. These sampling techniques were partly driven by the study’s conceptual framework, which sought to aid the exploration and discovery of what inhibits or promotes access to, and use of, skilled maternal health services among Muslim women. The selection of participants was based on a number of pre-set inclusion criteria: ease of recruitment, participant’s availability and willingness to participate in the study, and the ability/capacity of a participant to consent to participate in the research. As the emphasis of qualitative research is not always on generalisation hence randomisation might not be a necessary requirement, these sampling techniques were particularly appropriate [22].

The actual recruitment process involved advertising the study at local mosques in the three study communities via religious leaders and Community-based Surveillance Volunteers (CBSVs). CBSVs are recruits from local communities who have been trained by the District Health Management Team in various aspects of community health, including but not limited to reporting the outbreak of diseases as well as births and deaths in their communities. All Muslim women who were either pregnant at the time of this research or had given birth between January 2011 and May 2012, were invited to participate in the study. The CBSVs then helped the researcher to recruit interested individual participants for interviewing. Having grown up in the study communities, the CBSVs were very conversant with the local dialect and cultural nuances and were therefore in a good position to advise the researcher on suitable participants, arrange meetings, and negotiate solutions to potential and actual problems. In Buipe for instance, the researcher had wanted to conduct interviews within one particular week. However, upon discussion with the community CBSV, it was realised that on two particular days within that week, interviews could not be effectively conducted. This was because one of the days was the local community market day - a day that most women are expected to sell their farm products or buy things that will be needed for the week. The other day was a Friday – a day that most Muslims are expected to congregate at Mosques to pray.

### Data collection

Focus group discussions (FGDs) were the main data collection methods. This data collection technique was adopted partly because of its practical relevance in helping to reproduce Muslim women’s experiences of seeking skilled maternal health services in a normal peer-group interpersonal exchange. But the choice was also based on the methodological literature. According to Morgan, because FGDs have the ability to ‘give a voice’ to participants, they should be used where there is the need to identify participants’ perspectives and frames of meaning [23]. Green argues that a group setting often works well for generating talk about health, and that FGDs provide broader views about health and illness meanings [24]. Because FGDs in this study were interactive, participants were able to query and challenge each other as well as explain themselves; hence offering validated data on the extent of consensus or diversity.

In all, 6 FGDs – two in each study community - were completed. Women in groups were segmented by age (15–30 years, and 31–45 years) because initial discussions with CBSVs suggested that there were age hierarchy conflicts among women in the study communities. In other words, younger women (15-30years) were not likely to freely express their views in the presence of older women (31-45years) because of cultural norms, which require young people to listen to older people. Segmenting discussants by similar age groups contributed to making participants more confortable when expressing their opinions or sharing their experiences within the group context. To further limit the effect of any participant dominating the discussion, all participants were constantly encouraged, especially the quieter ones, to speak, share their opinions as well as agree and disagree with others where they felt the need to do so. In addition, themes and issues raised and discussed during FGDs were summarised and orally presented to participants to confirm, alter or reject at the end of the discussion. This was to make sure that the information collected accurately represented most participants’ views.

Groups consisted of 9–12 participants. Discussions in the focus groups lasted 2.5 to 3 hours, and ended when a point of saturation was reached (i.e. when no new issues seemed to arise). All FGDs were held in the study communities at communal places such as the premises of local community Mosques. All discussions were held in the local dialect – *Gonja*. This was done because participants’ fluency in English was very low. Because the author’s knowledge of the interview language was limited, the CBSVs were trained and engaged to facilitate the discussions. While all the CBSVs were trained prior to the interviews, it is possible that some may not have asked some of the questions rightly or they may have been biased in the way they asked some questions. To address this the researcher engaged all the CBSVs in a continuous review of the questions and interview process as the actual data collection progressed. This was to make sure that the questions were appropriate and rightly asked, and that the questions were understandable to research participants.

To complement the FGDs, individual interviews (IIs) were also conducted with a random sample of the women who participated in the FGDs. The choice of the individual data collection technique was informed by previous arguments that people may not necessarily tell the truth in any objective sense when it comes to sensitive issues such as health within a group context [25]. In some of the FGDs for example, some women declined to tell their reasons for not accessing and using skilled maternal healthcare services at health facilities. They considered that those reasons and experiences were personal and preferred not to talk about them among their peers. Instead, they suggested that if the researcher wanted to know, they were happy to discuss those reasons and personal experiences privately with the researcher. In a number of instances where the researcher made a follow-up on such participants, it was discovered that the reasons often bordered on the actions and inactions of another person in the family (mostly husbands and mothers-in-law), for which reason these women felt that openly discussing them may threaten the stability of their family. For this reason, FGDs were triangulated with IIs. A major difference between results from the FGDs and those from the IIs is that while the FGDs generated communal experiences, the IIs produced individual level information which addressed sensitive issues such as personal experiences of childbirth and barriers to accessibility to, and utilisation of maternity care services. Also because the questions in the IIs were specific to individual participant’s experiences, participants were able to individually consider the questions more widely and develop appropriate independent ideas and responses. This provided IIs with a built-in capability to challenge the researcher’s own preconceptions, and enabled participants to answer questions according to their own frames of reference.

The selection of women for the IIs involved a number of steps. To begin with, all the participants in each of the FGDs were assigned numbers, starting from one. These numbers were then written on small pieces of paper. Each of these pieces of paper was folded and mixed up with others in a calabash. One discussant was then chosen by the whole group to randomly select the required number of participants. The required number of participants was predetermined at 5% of the total number of women in each FGD. Sampling 5% of the women in each group yielded sufficient numbers of participants whose individual views and experiences could be explored in-depth to triangulate views and experiences already gathered in the group context. Each of the randomly selected women was then invited again to participate in the IIs. None of them refused the invitation. Separate individual informed consent was obtained from each participant. Except three cases, all IIs were conducted in the homes of the women.

While acknowledging that the adoption of the simple random sampling approach to the IIs clearly contradicts the rejection of random sampling for the FDGs, it was necessitated by the fact that almost all the participants in the FGDs also expressed their interests in taking part in the IIs. Due to both time and resource constraints, it was however not possible to individually interview all interested participants. Randomisation was therefore used as a pragmatic and ethical strategy to ensure justice, fairness and transparency in the sampling procedure. Thus it ensured that the selection of participants for IIs was purely due to chance. Indeed, the idea of chance - which was embedded in the sampling procedure - helped to eliminate questions about why one woman was included and another excluded from the IIs.

In total, 45 IIs were completed with individual Muslim women. Interviews lasted 20 to 30 minutes. These interviews were relatively shorter because each interview focused on the specific experiences of individual Muslim women, and explored very specific questions in relation to individual experiences. All interviews were conducted in *Gonja.*

### Instruments

An open-ended thematic topic guide was the main research instrument. The instrument was designed to ensure that similar themes and questions were covered in each discussion or interview. The instrument however had built-in flexibility that allowed for any pertinent but unexpected issues that arose during the interview process to be further probed. The instruments focused on exploring Muslim women’s maternity care needs, experiences of seeking or not seeking maternity care services, women’s interaction with maternal healthcare service providers, and the barriers and enablers of access. To ensure reliability, the instrument was pre-tested in one of the three study communities. This helped to reframe questions, clarify and use more appropriate or easily understandable concepts. All discussions and interviews were audio tape-recorded alongside hand-written field notes.

### Analysis

Data were analysed using the Attride-Stirling’s thematic network analysis framework [26]. The Attride-Stirling thematic network analysis framework is a method for conducting thematic analysis of qualitative or textual data, which allowed for open and methodical discovery of emergent concepts, themes and relationships through the application of principles of inductive reasoning to generating themes while also employing predetermined (deductive) code types to guide data analysis and interpretation [26]. Although this approach to qualitative data analysis shares common features with some of the more standard thematic analysis, it is unique in that it provides a practical and systematic technique for breaking up text, and finding within it explicit rationalisations and their implicit signification [26]. In particular, the approach’s focus on networks allows for the exploration of different themes and the interconnections within and between themes. This data analysis technique was chosen to reflect the conceptual framework outlined above, which aims not only to describe Muslim women’s maternity care needs and experiences but also to explore the barriers to, and facilitators of, access and use of maternal health services in health facilities.

The actual data processing and analysis process involved several steps. Following the completion of interviews, a *Gonja* language specialist was contracted to transcribe all tape-recorded interviews. Three Gonja language experts other than the one who did the initial translation from Gonja to English then performed back-to-back translations into English. The aim here was to verify the accuracy of the translations. The author then immersed himself in all transcripts and interview notes through reading and reviewing. This first step was completed with separate summaries for each transcript outlining the key points participants made. All transcripts were then exported into NVivo 9 qualitative data analysis software, where the data ware both deductively and inductively coded. Data coding continued until theoretical saturation was reached (i.e. when no new concepts emerged from successive coding of data). The completed code structure was applied to develop and report themes. Themes simply represented some level of patterned response or meaning within the data set [27]. In total, 37 codes were identified. These were grouped into 9 basic themes that were further clustered into 7 organising themes, and 2 global themes in line with the Attride-Stirling’s thematic network analysis framework (Table 3). These global, organising and basic themes form the structure of the findings and discussion sections of the paper. To ensure that the themes reflected the data, the data segments related to each theme were thoroughly examined. Where necessary, refinements were made. Where appropriate, verbatim quotations from interview transcripts were used to illustrate relevant themes. The BioMed Central’s qualitative research review guidelines (RATS) were used to guide the analysis and reporting of results of the study.

**Table 3:** **Thematic network analysis framework (from codes to global themes)**

**Codes**	**Basic themes identified**	**Organizing themes**	**Global themes**
-There is joy in pregnancy and childbirth.	1. Pregnancy and childbirth are fulfilling biological functions women perform	1. Pregnancy and childbirth is role fulfilment, self actualization and empowerment	**Muslim Women’s experiences of pregnancy and childbirth**
-Women feel accomplished in giving birth safely.			
-A woman can die while pregnant or giving birth	2. Pregnancy and Childbirth is dangerous – you either die or live	2. Pregnancy and childbirth can be a dangerous event	
-Pregnancy and childbirth is an anxious phase of a woman’s life			
-Pregnancy makes women highly dependent on others			
-A pregnant woman needs care, love and empathy to be able to deliver safely	3. Care during pregnancy is important for safe delivery	3. Muslim women want skilled attendance at birth	
-Women should go to hospital when pregnant	4. Hospital delivery is good		
-It is good to deliver in a hospital			
-Midwives can help to deliver women safely			
-Muslim women are required to preserve bodily sanctity	5. It is a religious duty in Islam for women to preserve bodily sanctity	4. Religious obligation to maintain bodily sanctity limits Muslim women’s ability to access skilled care	**Barriers to accessibility and utilisation of skilled maternal healthcare services by Muslim women**
-Muslim women must dress properly			
-Muslim women must cover up their bodies			
-Covering is one of the religious rules and duties in Islam			
- People who bear no relationship with Muslim women must not see their nakedness			
-There is no privacy in hospital birth	6. Muslim women values privacy in health facilities when accessing maternal healthcare services	5. Lack of privacy in health facilities is a disincentive for Muslim women’s use of skilled care	
-Privacy is not given attention in health facilities			
-It is difficult to fulfil Islamic requirement to preserve bodily sanctity			
-There is privacy in homebirth but not in hospitals			
-TBAs usually cover the perineal area of a labouring woman’s vagina			
-Caregivers do not ask how Muslim women feel	7. Muslim women want respect when receiving maternal healthcare services	6. Healthcare providers’ insensitivities to Muslim women’s needs and concerns limit their access to skilled care	
-Nurses are disrespectful			
-Caregivers disrespect and disregard Muslim women’s preferences and cultural values relating to pregnancy and childbirth			
-Caregivers do not take Muslim women’s religious and cultural needs into account			
-Maltreatment and scolding is dehumanizing			
-Caregivers want women to obey instructions without question			
-Caregivers must respect their clients			
-Circumcision of male infants is a requirement in Islam	8. Culturally and religiously inappropriate care and health information does not promote effective communication between caregivers and Muslim women		
- Information on circumcision is usually unavailable in health facilities			
-There is poor communication between women and caregivers			
-Caregivers lack understanding of Muslim women’s religious needs			
-Muslim women alone do not make decisions regarding access to skilled care	9. Muslim women lack decision-making autonomy and depend on other people to making decisions regarding use of skilled care services	7. Muslim women’s lack of decision-making autonomy constrains their access to, and use of skilled maternal healthcare services	
-Urban women participate more in decision-making			
-Women with secondary or higher participate more in decision-making			
-Husbands are important decision-makers			
-Mothers-in-law play crucial roles in decision-making			
-Women must be submissive			
-Women must be obedient			

## Results

Muslim women’s accounts in relation to their maternity care needs, care experiences, and barriers to service use converged on a number of common themes, which are explored below.

### Childbirth and Muslim women’s maternity needs and care experiences

Discussions and interviews with Muslim women showed that they valued safer childbirth for a number of reasons. Most of these women live in societies where patriarchy and polygynous marriages are common. It was therefore reported that a woman needed to have children to ensure the perpetuity of her husband’s lineage and to enhance her own bargaining power in the relationship.

Several women, particularly rural women and women with no formal education reported that successful childbirth not only brought them honour but also guaranteed them a place in the rather competitive polygynous environment. For this reason these women said that a pregnant woman needed care, love and empathy from both family members and healthcare providers to be able to give birth safely. This, together with fears that a woman might die in the process of giving birth, explains why these women desired skilled care during pregnancy or delivery. Although there were sporadic speculations during FGDs about the existence of few fundamentalist Islamists who completely eschew western medicine, the Muslim women who participated in this study acknowledged the importance of skilled maternity care. Indeed the majority of women did want professional assistance in a health facility setting where their maternity needs could be met.

*“I believe it is important for every pregnant woman to go to hospital. I am saying this because of my own experience. When I was pregnant, I never went for check-up for about seven months. But some friends advised me to go to hospital and check if everything was fine. I went and when the doctor had finished examining me, he said my blood level was low [anaemic] and that this could affect my baby. I was then given blood and the doctor advised me to eat well especially egg, meat, beans, and vegetables. I believe it is because of this that I didn’t suffer much during labour. Maybe if I didn’t go to hospital I could have died or I couldn’t successfully give birth. That’s why I think it’s good for all women to go to hospital when they are pregnant”* (Lactating Mother, FGD).

Several of the accounts these women gave suggested that their birthplace choice was rapidly shifting from the home towards formal healthcare institutions where skilled birth attendants were likely to be available. Indeed, available health facility level data from the Central Gonja District Health Directorate largely corroborated the above accounts. In Buipe for example, skilled delivery rose from 18.1% in 2009 to 22.2% and 36.1% in 2010 and 2011 respectively [17]. During the same period, skilled delivery in the entire district rose from 13.4% in 2009 to 14.4% and 24.3% in 2010 and 2011 respectively. Despite the fact that many participants expressed their preference for skilled attendance, it was reported that, in practice, a number of factors and considerations, which were related to their religious beliefs, significantly hindered their ability to access and use these services. These factors are discussed below.

### Islam, female bodily regulation, and access

Several personal narrative accounts from participants in both rural and urban settings indicated that the Islamic religious faith impacted Muslim women’s ability to access maternity care services through what participants approximated as ‘the duty of maintaining the sanctity of the female body in Islam’. Basically, this duty enjoins Muslim women, especially those who have entered into properly constituted marriages or unions in accordance with Islamic law and practice, to preserve their physical body away from the prying public eye, particularly from the opposite sex with whom they bear no intimate relationship. This obligation is often fulfilled through practices such as proper dressing and covering up most parts of the body, although hands and face are generally considered acceptable if uncovered.

*“The reason why we Muslim women don’t go to the hospital to give birth is that, if you go there they will see your body…your nakedness and private parts. As Muslim women, it is not proper for people, especially men, to see us naked just like that. It is not proper Muslim behaviour. We are supposed to dress well by covering our bodies. If you are married, the only man who should see your nakedness is your husband. But as you know, in some hospitals there are men in the place where women go to check their pregnancies or even in the rooms where we are suppose to give birth”* (Pregnant Woman, FGD).

Another participant related:

*“When I was pregnant with my first child, one day I went to the clinic for antenatal. The midwife told me to go into one room so that she can examine me. When I went into the room, there were two other nurses in there…one was a man. But the midwife asked me to remove my dress and lie down. But the man was there; so I said no I’d wait until the man goes out, because as a Muslim, it is improper for a man who is not my husband to see my nakedness. The nurse was angry and started speaking abusive words…me too I became very annoyed and walked out of the room. I never went back there again till I delivered, and I will never go there if I get pregnant again*” (Lactating Mother, FGD).

One participant also said:

*“Are you asking me why I didn’t deliver at the clinic? Well, I didn’t because I didn’t want any stranger, especially men at the clinic to see my naked body. As a Muslim, it is not proper to expose my body to just anybody…I believe I’m not the only one…I know some pregnant women in this community who want to go and check their pregnancy or even give birth at the clinic, but they fear that maybe a male nurse, midwife or doctor will be there or will be made to perform operation on them. Because Allah - the greatest, the merciful and the benevolent - does not like us to leave our bodies open just anyhow, we feel it is not proper for the doctor to see us naked. Even our husbands would not be happy if that happens*” (Lactating Mother, II).

Several participants noted that covering up their bodies was one of the many religious rules and duties in Islam, and Muslim woman who wanted to practice the Islamic religious faith must observe this rule. By contrast, these women reported with satisfaction the fact that in homebirths, traditional birth attendants (TBAs) and other family members who were usually all women, sometimes avoided seeing the labouring woman’s vagina while delivering the baby. Accordingly, the perineal area of the labouring woman was covered using a piece of cloth. For them, this was very consistent with the requirement to uphold the sanctity of the female body in Islam. In this regard, some Muslim women reported that it was sacrilegious to either undress before a total stranger like a nurse or midwife or allow a non-Muslim male doctor to attend their delivery. Several participants in communities such Buipe and Mpaha where there were healthcare facilities, expressed their strong preference for having a female birth attendant during labour or delivery, and suggested that doctors and midwives could incorporate this into their practice so as to address their concerns. Others also recommended religious and cultural matching of patients such that Muslim women could be matched unto female caregivers or caregivers who share the Islamic faith.

#### Islam, privacy, and access

Related to the modesty requirement for Muslim women to maintain bodily sanctity was the issue of privacy in maternity hospitals. Several of the women interviewed reported that under the current maternal healthcare regime in Ghana, hospital and clinical structures and practices made it extremely difficult for them to maintain privacy and discuss their health concerns with nursing staff. This was a disincentive for many Muslim women who needed skilled maternity care.

*“The reason why we the Muslim women feel very reluctant going to the hospital to check our pregnancy or give birth is because it is very difficult to prevent other people from seeing us or hearing our problems there…if you give birth in the hospital, you have to share the ward, toilet, bathroom and other things with other patients who might not be Muslims. Sometimes, how to get a quiet place to even pray is difficult. This makes it hard for us to observe all our religious duties. But at home, we can get up and go to Madam Hawa [a community TBA] to check our pregnancy and nobody will know. If we give birth at home too, everything is private; we can observe all our prayers and do all the necessary things without anybody knowing. In the hospital, it is difficult to do all these things”* (Pregnant Woman, II).

One participant also said:

*“I didn’t deliver at the hospital because the first time I gave birth at the hospital…it was not too good for me. I didn’t like the way the place was open. There were other patients, nurses, midwives and doctors in the labour ward. So everyone could see you or hear what you are doing. It was difficult to even get a quiet place to pray…and anytime I had visitors and they needed a private or quiet place to pray, it was very difficult. This is very bad especially for those of us who are Muslims…you know we need our private space to pray and do other things…you know sometimes I feel that maybe my religion is a barrier, but if the health people can provide every patient their own delivery room, or even if they can use curtains to shield every patient, I think it will help. Also, it would be better if they create quiet places for prayers…this is what happens if you go to a Muslim hospital*” (Lactating Mother, FGD).

Despite the importance Muslim women attached to privacy, they noted that privacy was not usually ensured neither was it given any serious importance in maternity wards. Women reported that during physical examinations, pregnant women were often palpated with open doors or curtains, which made it easy for other patients or even male healthcare workers to see the women. Several participants said this was very concerning because it made it impossible for them to fulfil their religious requirements to preserve bodily sanctity. Similarly, women said that case histories and clinical examination of pregnant women often took place in the midst of other patients. This was made worse by nurses’ tendency to interview women in loud voices. This made it easy for other patients to hear the health problems or concerns of other women. While most of the women acknowledged that limitations imposed by infrastructural constraints might be contributing to this situation, it was clear from their accounts that lack of privacy in ANC clinics and labour wards, and the way in which caregivers ignored this crucial aspect of care, constituted an important factor negatively affecting Muslim women’s maternal healthcare-seeking behaviours.

### Healthcare providers’ insensitivity and access

Several of the women interviewed underscored the importance of healthcare providers having an understanding of, or at least, familiarity with and respect for their [women’s] religious beliefs, practices and maternity needs. However, these women said that most midwives and nurses, especially non-Muslim ones, often seemed uninformed about the religious obligations, practices and maternity needs of Muslim women. Being uninformed about Muslim women’s maternity care needs and religious and cultural practices, it was reported that some caregivers were unable to provide knowledgeable healthcare, guidance and information that took Muslim women’s needs into account. Even where healthcare providers were well aware of the religious practices and maternity care requirements of Muslim women, it was reported that such caregivers did not always take Muslim women’s concerns seriously, and that in most cases such caregivers became angry any time women asked for their religious or maternity needs to be acknowledged and respected. For instance, some participants who have had prior experience of health facility birth reported that on several occasions caregivers ignored their maternity care needs or completely disrespected their religious practices, actions that made them ‘felt bad’.

*“Me I have said that I will not deliver in a hospital that has no Muslim doctors or nurses. This is because nurses who are not Muslims do not usually know that there are certain things that we Muslim women are suppose to observe. When I was pregnant with my first child, I delivered in the hospital, but my experience was bad…there were so many male nurses entering our ward. I asked them if they could always knock on the door before they enter so that I can dress and cover myself properly. But they didn’t respect it…one of the male nurses even came into the ward when I was half naked…I was very upset…the worse thing was that some of the nurses started making fun of me…they said perhaps it is because my body is ugly that’s why I didn’t want any body to see. I really felt bad …they didn’t understand that as a Muslim woman, it is not proper for any man to see my nakedness*” (Pregnant woman, II).

Some participants also said that giving birth in a health facility setting often came with other challenges such as how to obtain appropriate food (i.e. *halal* meals) especially in situations where the health facility was far away from home or from relatives and friends. This problem was widely reported in the rural community of Tidrope where there was no healthcare facility, and where utilisation of maternal healthcare services often involved travelling for a relatively longer distance away from home. Unfortunately, it was reported that some healthcare providers did not understand the importance of this dietary requirement to Muslim women. One participant related her experience thus:

*“I didn’t deliver my baby at the hospital…I didn’t because the first time I got pregnant and was sent to Kintampo hospital to deliver, I had several problems like how to get food. You know I am a Muslim…our food should be halal, but because I was far away from home and my family, it was difficult to make sure that my food was halal. I was relying on the food that my husband brought to me daily, but you know my husband was coming from a very far place, so sometimes I got hungry before he came with the food…one day I was so hungry but my baby too was crying so I begged one of the nurses to buy food for me. When she brought the food, I asked her whether the food was halal, but she said she did not know…she even became angry and threw the food at me. She said if I didn’t want the food then I should go and buy myself… I felt bad. She did not understand that I must not eat any food that originates from pig or contain alcohol. So I have told myself that I will never go to that hospital*” (Lactating Mother, II).

Other participants reported that, although circumcision of male infants was a requirement in Islam, information relating to this service was usually not available in most health facility settings, especially in rural areas. This served as a disincentive for some Muslim women to give birth in a health facility.

#### Muslim women’s decision-making autonomy and access

One other major reason why Muslim women in northern Ghana are unable to use skilled maternal health services relates to their decision-making autonomy within the family set-up. In FGDs and individual interviews, women reported that although they were often expected to nurture their pregnancies and successfully give birth, the power to make decisions regarding how and when to seek pregnancy and birthing care was dispersed among a complex network of actors, with husbands and mothers-in-law being seen to have the greatest share of authority as final decision-makers.

*“I think one main reason why we Muslim women in this village don’t go to deliver in the clinic is that many of us are powerless. This is because as Muslim women, we are expected to submit ourselves to our husbands…so some of us depend on our husbands to make decisions for us. So if my husband makes a decision that I shouldn’t go to give birth at the hospital, I have to obey him…you know both the Koran and Hadith emphasized that obedience and submissiveness are marks of good womanhood”* (Pregnant Woman, FGD).

Another participant reported her experience:

*“I believe the reason why some Muslim women don’t go to hospital to give birth is because of their husbands. I say this because when I was pregnant I wanted to go to hospital to deliver but my husband said no…he said none of his other two wives had given birth in a hospital before. So I had no option. I didn’t want any problems with him. Besides, if I had gone to the hospital, my other co-wives will make fun of me. They will say I’m weak that’s why I went to hospital to give birth…you know my husband can end our marriage just because of this”* (Lactating Mother, FGD).

One woman also said ‘you see part of the reason why the women are not going to hospital to give birth is because they depend on other people like their husbands to make that decision’ (Lactating Mother, II). Indeed, many of the accounts these women gave suggested that a patriarchal ideology, coupled with low levels of female education, high levels of economic marginalisation, and traditional interpretations that emphasize passages in the Koran and Hadith that define women as distinctly submissive, obedient and subordinate to men, had created a social, economic and political environment in which women were dependent on men. Although some participants acknowledged that this situation was changing especially in urban settings and among the younger generation as well as women with at least middle and secondary education, they reported that a combination of machismo, the culture of female submissiveness and women’s economic dependence on men still created an unequal power relationship between men and women. In this unequal power relationship, women often ceded their autonomy and decision-making power to men, including decisions concerning access to, and use of, maternal health services.

Despite the above, a few of the accounts from women who had some level of formal education indicated agency and defiance or at least the potential for defiance in situations where they needed or wanted skilled care but their husbands or mothers-in-law had decided against using health facility services.

*“When I was first pregnant, my husband and mother-in-law didn’t want me to go to hospital to check my pregnancy. But inside me I felt like I should go because some of my friends were going all the time, and any time they go and come, they tell me a lot of things that the doctors and midwives ask them to do or not to do. I realized that the things my friends were telling me were very helpful, so one day I told my mother-in-law that I must go to see the midwife whether they like it or not, and I went. And I’m very happy that I went because the doctor gave me a lot of advice, which helped me to deliver my baby well*” (Lactating Mother, FGD).

Other women, many of whom had some middle and secondary school education, also reported that they found an influential person in the community or a bosom relative or friend to plead with their husbands to allow them [women] to go to the hospital to give birth.

## Discussion

In recent years, Ghana’s government has made considerable progress in making maternal healthcare services more accessible to women. Despite this, Muslim women remain one sub-population in the country among whom utilisation rates of health facility birthing services are very low [2]. The purpose of this qualitative study was to explore the maternal healthcare needs and care experiences of Muslim women and the barriers Muslim women faced in accessing and using maternal health services in northern Ghana.

The paper documented several interrelated themes that interacted in very complex fashion not only to trigger Muslim women’s need for skilled birthing services but also several barriers to utilisation of services. For instance, findings suggested that Muslim women in northern Ghana valued safer childbirth because it ensured the perpetuity of their lineages, enhanced their bargaining power in their families, brought them honour, and guaranteed their position especially in polygynous marriages. Coupled with the related theme of fear that a woman might die in the process of giving birth, the majority of the women interviewed in this study expressed their preference for professional assistance in a health facility setting where their maternity needs were likely to be met. The finding that many women wanted skilled assistance during childbirth clearly has policy implications. It suggests that Muslim women might be willing to access and use skilled birthing services in health facilities if the services are organised and delivered in a way that is responsive to their maternity needs and care expectations.

Notwithstanding the fact that the majority of the Muslim women interviewed in this study wanted professional assistance during pregnancy or childbirth, findings from focus group discussions and individual interviews revealed that aspects of their religious beliefs and practices made it extremely difficult for them to access and use skilled maternal health services designed to serve the general population. These difficulties arose from a number of specific factors including, a religious obligation to maintain bodily sanctity through modest dressing and the avoidance of unlawful bodily exposure or contact with certain people including male caregivers, lack of privacy in healthcare facilities, healthcare providers’ insensitivity and lack of knowledge about Muslim women’s religious and cultural practices, ineffective communication resulting from health information that lacked the cultural and religious specificity to meet the unique maternity needs of Muslim women, and Muslim women’s limited decision-making power within their families. Together, these factors acted to limit the ability and willingness of many Muslim women to access and use skilled birthing services at health facilities despite government efforts to ensure that all births take place in a health facility under the supervision of a trained health professional.

It is important to point out that the barriers that Muslim women in northern Ghana face in accessing and using skilled maternal healthcare services are not unique. Previous studies in England [28], New Zealand [29], and Canada [13] have documented similar barriers among Muslim women. Indeed, it is also worth pointing out that some of these barriers such as the desire for privacy and the issue of Muslim women’s dependence on husbands and relatives in relation to maternal healthcare utilization decision-making are also being experienced by non-Muslim women as shown by some recent studies in Ghana [3,4]. However, the Muslim women sampled in this study expressed specific needs as well as faced specific access barriers, which were directly related to their religious beliefs and practices. These findings are therefore very novel in the context of Ghana where previous research have failed to highlight the unique maternity care needs of Muslim women and the specific barriers they face in accessing and using maternal health services designed to serve the general population.

Most of the women interviewed strongly wanted a female attendant at birth. Others wanted to be attended to by a healthcare provider who either shared the same religious faith as theirs or had a better appreciation of the unique religious practices and requirements of Muslim women. Some also wanted male circumcision services within health facility settings to enable them fulfil their religious obligation to circumcise their male infants within a reasonably good time after delivery. Several of the women also wanted privacy in maternity wards as well as quiet prayer rooms, while many others expressed discontent over the attitude of healthcare providers and many of the procedures healthcare providers carried out during ANC clinics and labour, which rarely conformed to their religious practices or promoted their dignity. Unfortunately, as many of the women reported, current hospital and clinical structures and practices made it extremely difficult for these needs to be met. This acted as a disincentive for many Muslim women who desired to access and use skilled maternity care services in health facilities.

That Muslim women’s maternity care needs are not being met under the current maternal healthcare regime in Ghana directly calls for changes and improvements in nursing, midwifery and obstetrical/gynaecological practices. For instance contrary to public health definition of skilled attendance at birth, women emphasised respectful care (i.e. care, love and empathy for pregnant women), a practice that is currently promoted. Changes in nursing, midwifery and obstetrical/gynaecological practices must therefore include the creation of an enabling environment within healthcare facilities to address privacy issues, training and retraining of healthcare providers to provide culturally competent and patient-centred care, as well as improvements in provider-client communication. In effect, there is the need for changes at the health facility level that create a diversity responsive maternal health service [13,30]. For example, healthcare facilities would be more responsive to Muslim women’s needs if organisational procedures were put in place to match female birth attendants to Muslim women. Again, the maternal healthcare system in Ghana would be more responsive to the religious needs of Muslim women if current and future designs of health facilities were to incorporate quiet prayer or worship rooms. In this regard, it might be useful for the Ghana Health Service and Ghana’s Ministry of Health to learn from the design and organisation of Islamic hospitals in the country since several of the women interviewed in this study said their religious and maternity care needs were often met in these hospitals. Similarly, maternal health services would be more responsive to the needs of Muslim women if procedures were put in place to enable Muslim women to be matched unto physicians who are also Muslims or who are, at least, familiar with the religious practices and requirements of Muslim women.

Of course, given the limited financial and human resources that are usually dedicated to healthcare provisioning in Ghana, implementing the above recommendations can put additional pressure on the healthcare system. However, the adoption of new practices such as religious or cultural matching can reduce the *social distance* (i.e. differences in culture or religion) between maternity caregivers and Muslim women. As one previous study in Ghana has shown, women preferred to travel further and face higher opportunity costs to see providers who were the same ethnic or religious group as themselves [31]. The study revealed that this was because a provider from the same ethnic or religious group was perceived as having a smaller social distance and therefore worth travelling the extra distance for. In addition, it is essential that efforts aiming at changing, modifying or accommodating some of these religio-cultural norms and practices should involve community-based health education campaigns. Such campaigns should focus on rural communities such as Tidrope where there are no health facilities, and communicate both the importance of women delivering their babies with a relatively well-resourced skilled health professional in attendance, and challenge those aspects of traditional cultural practices and Islamic religious beliefs and practices that constrain women’s ability to access life-saving maternal healthcare services. In this regard, religious leaders such as local community Imams – who usually wield considerable power and influence at the community level - could be involved to lead education campaigns that seek to encourage more Muslim women to access and use skilled birthing services.

Apart from the fact that Muslim women’s maternity care needs are not being adequately met within health facility settings, focus group discussions and individual interviews with women also revealed that in many instances husbands were critical decision-makers. Similarly, mothers-in-law, whose own experiences of pregnancy and childbirth might have involved limited access to skilled care, and who may put greater value on modesty and other cultural requirements, discouraged their daughters-in-law from seeking care from trained providers. As reported by several participants, the power-play between women, their husbands, and their mothers-in-law often resulted in women either not accessing needed care or reporting to a health facility only when complications have set in. In this regard, patriarchy, gender inequality and economic marginalisation of women were factors that inhibited Muslim women’s ability to autonomously make decisions about access and use of birthing services. This suggests that efforts and strategies that help to improve women’s economic status and promote gender equity might have potential benefits for increased and equitable access to maternal health services in the study communities. This, of course, is not to say that Muslim women’s enjoyment of equal decision-making rights in the study communities will automatically result in increased access, neither is it suggesting that non-egalitarian gender ideologies necessarily correlate with worse maternal healthcare access for women. Also, while it is important to improve women’s bargaining power in the household, it is equally important that efforts are made to directly engage men and mothers-in-law on maternal health issues in the study communities. Promoting Muslim men’s involvement in issues of maternity care could be particularly useful because many women in the communities where this research was conducted still required the permission of a husband or a male partner to pursue activities outside the home, including attending antenatal clinic or giving birth in a healthcare facility. Strategies for involving men could include couple counselling as well as the provision of male-friendly maternity clinics and services. While patriarchy, religious and cultural perceptions about the role of men as bread winners might hinder men’s effective involvement in matters of maternal health, programmes that promote Muslim men’s involvement in the study communities could be particularly vital in increasing men’s understanding of the relevance of skilled attendance at birth. This can enable men to play more supportive roles in the area of maternal healthcare access.

Put together, the findings in this paper highlight the relevance of few religious rules and duties in Islam that could affect the low use of healthcare services. The findings clearly suggest that the use of the religio-cultural thesis as a conceptual framework in this paper was most appropriate in drawing attention to the Islamic religion as a critical factor that constrains access and use of modern maternal health services among Muslim women [16]. The use of the appreciative inquiry approach particularly enabled Muslim women to identify not only factors that inhibited access to and use of skilled maternal health services, but also to search for solutions that already existed or that could be used to promote access to and use of maternal health services within Muslim communities. By discovering both positive and negative attributes, stories, and experiences of Muslim women, the findings of this study offer opportunities for stimulating core positive change and re-examining present problems in ways that enable problem solving. Of course, Muslim women are not a homogeneous group; but are members of different ethnic groups who might belong to different schools of Islamic jurisprudence or be at different levels of socio-economic development and empowerment. Therefore, the impact of few rules in Islam on gender and reproductive choices could largely be a function of the socio-political context in which these issues are defined [32]. For example, it is possible that the low level of education among the women interviewed for this study could have impacted negatively on their ability to access and use skilled maternal health services. Maternal education has been found to be positively associated with access and use of many of the elements of skilled maternity care such as delivering in a hospital [11,33]. Influences of maternal education on maternal healthcare access can be effected in several ways, including improving the ability of women of reproductive age to produce good maternal health outcomes without even relying on health services by influencing their reproductive behaviours such as contraceptive use, and empowering women to be able to leverage decision-making regarding reproductive choices and access to birth services within the household [34]. Indeed, many of the women in this study who were able to leverage decision-making or defied the decisions of their husbands or mothers-in-law were women with some form of education. Majority of such women were also living in urban areas. The findings and discussion here therefore suggest the need for improvement in women’s educational status up to at least secondary level if equity in access to skilled maternal health services is to be achieved and the maternal health-related MDGs attained in Ghana.

The findings and recommendations in this paper should however be read against the backdrop of certain limitations. The research reported in this paper was conducted in only three communities in the Central Gonja District of northern Ghana. While focusing on a small number of communities enabled greater in-depth understanding of Muslim women’s maternity needs and the access barriers they faced, the limitation of applying the findings to other parts of the country is acknowledged. This is more so because Muslim women are not a homogenous group, hence the perspectives of those interviewed in the Central Gonja District might be different from what pertains elsewhere in Ghana. In addition, much of the data was self-reported, and collecting data through recall of reproductive history generates information that is liable to recall bias. Also the basis on which the study participants were identified and recruited could potentially have introduced biases into the recruitment process. For instance, advertising the study at local mosques could potentially exclude women who did not patronise such local mosques. Similarly, the use of CBSVs to recruit interested participants could also have introduced some bias in the recruitment process. More over, 20–30 minutes is a relatively short time for an in-depth interview to allow each participant enough space to fully articulate their subjective lived experience. Finally, the results presented in this paper do not cover the perspectives of healthcare providers but only essentially give an account of the interaction between cultural preferences of Muslim women versus competitively fairly secular service organisation and delivery embodied through individual health workers. To explore multiple and balanced perspectives on the issues, future research could triangulate the information from Muslim women with interviews with healthcare providers. These potential limitations notwithstanding, important lessons could be drawn from the findings in this paper to inform policies that seek to encourage Muslim women to use skilled maternity care services in other parts of the country and beyond. In particular, the results of the study provide useful pointers for Islamic societies and organisations, and healthcare providers in Ghana and across the world, to participate in providing technical expertise in the delivery of acceptable healthcare to Muslim women in accordance with Islamic religious beliefs and practices.

## Conclusion

This study has indicated that Muslim women in the Central Gonja district have specific maternity care needs as well as face unique barriers to healthcare access. Therefore, maternal healthcare services designed to meet the needs of mainstream Ghanaian women might lack the flexibility and responsiveness to meet the unique religious needs of Muslim women. There is therefore the need for Ghana to move beyond free maternity care to engage with the specific maternity care needs of all women and address the unique cultural constraints women face. Attention must be given to addressing some of the barriers that routine nursing and clinical practices and structures present to Muslim women, as well as other social determinants of maternal health including women’s relative powerlessness in decision-making. Changes that address the unique maternity care needs of Muslim women and the access barriers they face have the potential to create a more inclusive and responsive maternal healthcare system that could ultimately improve maternal health outcomes in Ghana.
